# Single cell RNA sequencing for breast cancer: present and future

**DOI:** 10.1038/s41420-021-00485-1

**Published:** 2021-05-14

**Authors:** Lili Ren, Junyi Li, Chuhan Wang, Zheqi Lou, Shuangshu Gao, Lingyu Zhao, Shuoshuo Wang, Anita Chaulagain, Minghui Zhang, Xiaobo Li, Jing Tang

**Affiliations:** 1 Department of Pathology, Harbin Medical University, Harbin, 150081 China; 2 Department of Microbiology, Harbin Medical University, Harbin, 150081 China; 3 Department of Oncology, Chifeng City Hospital, Chifeng, 024000 China

**Keywords:** Breast cancer, Tumour heterogeneity, Breast cancer

## Abstract

Breast cancer is one of the most common malignant tumors in women. It is a heterogeneous disease related to genetic and environmental factors. Presently, the treatment of breast cancer still faces challenges due to recurrence and metastasis. The emergence of single-cell RNA sequencing (scRNA-seq) technology has brought new strategies to deeply understand the biological behaviors of breast cancer. By analyzing cell phenotypes and transcriptome differences at the single-cell level, scRNA-seq reveals the heterogeneity, dynamic growth and differentiation process of cells. This review summarizes the application of scRNA-seq technology in breast cancer research, such as in studies on cell heterogeneity, cancer cell metastasis, drug resistance, and prognosis. scRNA-seq technology is of great significance to deeply analyze the mechanism of breast cancer occurrence and development, identify new therapeutic targets and develop new therapeutic approaches for breast cancer.

## Facts

- scRNA-seq technology is a potent tool to study cell heterogeneity, including normal breast cells, breast cancer cells, fibroblasts and immune cells.
- scRNA-seq technology provides a useful method to distinguish the molecular characteristics and investigate the mechanisms of breast cancer metastasis.
- The emergence of scRNA-seq technology provides more possibilities for in-depth exploration of drug resistance mechanisms and identification of novel therapeutic targets for breast cancer therapy.
- scRNA-seq technology can be used to identify markers to predict the prognosis of breast cancer patients.

## Open Questions

- scRNA-seq technology is a powerful tool to study single-cell biology, and it is powerful for studying the heterogeneity of cancer cells. Can it finally reveal the origin of breast cancer from stem cells or differentiating epithelial cells?
- Different treatment methods to the breast cancer, such as chemotherapy, target therapy and endocrine therapy, have been established based on the molecular subtypes, however, drug resistance to these therapies have been frequently observed in breast cancer patients. Can scRNA-seq reveal the molecular mechanisms of therapeutic resistance or failure in the breast cancer?
- Can the molecular characteristics of a single cell be transformed into molecular signatures based on bulk RNA expression profiles to predict the breast cancer treatment response or prognosis in the clinic?

## Introduction

Breast cancer is the most common malignant tumor in women with high recurrence and mortality^1^. Many risk factors, such as sex, age, estrogen status, family history, and unhealthy lifestyles, may augment breast cancer risk^2^. Breast cancer usually starts from atypical ductal hyperplasia and develops into benign tumors and even metastatic cancer under the constant stimulation of various carcinogenic factors, such as gene mutations, obesity, and the use of hormone therapies (progestin and estrogen), etc^2^. Two models, the cancer stem cell (CSC) hypothesis and stochastic model, have been developed to explain breast cancer occurrence and development^3,4^. The former holds that all tumor subtypes are produced by the same stem or progenitor cells. These cells undergo acquired genetic and epigenetic events, leading to different tumor phenotypes. The latter includes all tumor subtypes derived from any cell type (stem, progenitor, or differentiated cells). After accumulating sufficient random genetic events and epigenetic changes, it can gradually transform into a neoplastic cell. Although they disagreed concerning the origin of breast cancer, both models seem to explain the heterogeneity of breast tumor cells.

Many researchers have proposed that the heterogeneity of breast cancer is critical to diagnosis and treatment. The recognized molecular types of breast cancer are basal-like, luminal A, luminal B, human epidermal growth factor receptor-2 positive/progestrogen receptor negative (HER2+/ER–), and normal breast-like^5,6^. Currently, clinical therapies are based on the molecular subtypes and their features. For example, HER2-positive patients receive HER2-targeted antibody or small-molecule inhibitor treatment combined with chemotherapy, and patients with hormone receptor-positive or triple-negative breast cancer (TNBC) receive endocrine therapy (ET) and chemotherapy, respectively^7^. However, despite recent progress, this systemic treatment relying on the molecular classification of breast cancer has problems and poor efficacy for some patients due to drug resistance caused by the heterogeneity of cancer cells^8^. Regarding resistance mechanisms, several molecular mechanisms exist, such as increased drug efflux, drug target mutations, DNA damage repair, activation of alternative signaling pathways, and avoidance of cell death^9^. Epigenetic changes and the influence of the local tumor microenvironment are also considered crucial factors for drug resistance^10^. In addition, molecular and genetic heterogeneity is increasingly recognized in many tumors and can contribute considerably to drug resistance^11^. The emergence of single-cell RNA sequencing (scRNA-seq) technology can detect these heterogeneous individuals, decode the heterogeneity of breast cancer cells, refine molecular types, and open up new ways to overcome resistance^12^.

scRNA-seq is a new-generation sequencing technology based on nanopore equipment after second-generation sequencing was first developed in 2009. It has become a revolutionary tool to reveal the uniqueness of each cell and solve problems that cannot be answered by traditional techniques. scRNA-seq technology mainly includes the following processes: tissue dissociation to obtain a single-cell suspension, cell lysis, reverse transcription of RNA into cDNA, PCR amplification, high-throughput sequencing, and data analysis^13^. Many analytical methods for scRNA-seq are currently available, such as nanogrid single-nucleus RNA sequencing, the ISOP method, and UQ-pgQ2 combined with DESeq2 (Table 1). In recent years, scRNA-seq has been used to reveal the heterogeneity of cells, cell dynamic differentiation processes, tumor prognosis, treatment, and other aspects in cancer^14–17^. In this review, we will summarize the current application of scRNA-seq in breast cancer research and discuss the new progress of breast cancer heterogeneity and mechanism of breast cancer occurrence and development. This study will provide a theoretical basis and new ideas for clinical treatment and prognosis.

**Table 1:** Methods for analysis sc-RNA sequencing data in the breast cancer.

Algorithm name	Function	Ref.
Nanogrid single-nucleus RNA sequencing	Developed a high-throughput 3′single-core RNA sequencing method, which combines nano-grid technology, automatic imaging, and cell selection, and can sequence up to 1800 single-cores in parallel.	^55^
ISOP method	It provides a novel method to express the isoform level and heterogeneity in single-cell RNA sequencing data.	^56^
UQ-pgQ2 combined with DESeq2	It improves the analysis based on intra-group comparison and applies it to the public RNA-seq breast cancer data set.	^57^
Average-based approach (gene-level expression) to isoform abundance/splicing event	It highlights the importance of splicing mechanisms in defining tumor heterogeneity.	^58^
SSCA and SSCVA methods	It can recover known biological characteristics from the data set and the shallow sparse connection autoencoders used for gene set projection.	^59^
SCmutt	It is a new and reliable statistical method which identifies specific cells with mutations found in bulk cell data.	^60^
CSMF method	It can reveal common and specific pattern scenarios with important biological significance from interrelated biological data.	^61^
EVA	It is used for evaluating the heterogeneity of gene expression in pathways or gene sets in single-cell RNA-seq data.	^62^
Digitaldlsorter	The algorithm deep learning scRNA-Seq deconvolution gene expression data.	^63^
VDJView	It can mine and analyze single-cell multi-omics data.	^64^
DUSC	The system integrates feature generation based on deep learning architecture and model-based clustering algorithms to obtain compact and useful single-cell transcription data.	^65^

*CSMF* common and specific patterns via matrix factorization, *DUSC* deep unsupervised single-cell clustering, *ISOP* ISOform-patterns, *EVA* expression variation analysis, *SSCAs* shallow sparsely connected autoencoders, *SSCVAs* shallow sparsely connected variational autoencoders.

## scRNA-seq reveals the heterogeneity of breast epithelial cells

Breast cancer originates from breast epithelial cells and is caused by genomic alteration and loss of tissue homeostasis in breast epithelial cells^18^. Understanding normal breast epithelial cells helps to recognize the occurrence of breast cancer. Breast epithelium can form a ductal network structure that connects the nipple with a complex system comprising 12–20 lobules through a collecting duct and is embedded in adipose tissue. In the ductal and lobular system of the breast, the epithelium comprises two known cell types: inner secretory lumen cells and outer basal or myoepithelial cells. Recent reports have indicated that these two types of mouse breast cells are heterogeneous^19^. Karsten et al. isolated mammary gland epithelial cells from four developmental time points—nonbirth, 14.5 days of pregnancy, 6 days of lactation and 11 days after natural degeneration^20^. They found lineage differentiation of the breast epithelium via scRNA-seq technology. At the same time, common luminal progenitor cells in the luminal epithelium differentiated into intermediate restricted alveolar progenitor and hormone-sensing progenitor cells. Another study reported that breast epithelial cells are highly heterogeneous and divided into different subpopulations based on genetic markers^18^. scRNA-seq of normal breast epithelial cells from breast reduction surgery revealed three different epithelial cell populations: basal (KRT14^+^), secretory luminal1 (KRT18^+^/SLPI^+^), and hormone-reactive luminal2 (KRT18^+^/ANKRD30A^+^) cell types. These three epithelial cell populations can be directly associated with several established breast cancer subtypes, suggesting that different breast cancer molecular subtypes may originate from different cell subpopulations. This discovery contributes to understanding the initiation, development and pathogenesis of breast cancer. In summary, researchers found that normal breast cells are highly heterogeneous at the single-cell level. Reconstruction of the mammary gland epithelial cell growth trajectory via scRNA-seq technology further revealed obvious changes in the gene expression and biology of breast cells during development^18^. These studies are critical in understanding the correlation between different cell phenotypes in the mammary gland and breast cancer initiation.

## Application of scRNA-seq in confirming and validating the heterogeneity of breast cancer

### Heterogeneity of breast CSCs in breast cancer identified by scRNA-seq

The occurrence and development of breast cancer are inseparable from CSCs, which can self-renew^21^. The systematic analysis of CSCs is critical for understanding tumor progression and developing new treatment strategies. Chen et al.^22^ used a label-free algorithm to reveal the high-potency cell state enrichment of human breast epithelial cells using scRNA-seq data. The algorithm further predicts and proves that the stem-like state is bipotent and can differentiate into basal and luminal states. In addition, the bipotent stem cell-like state is associated with the clinical outcome, and the high expression of the Y-box binding protein 1 (YBX1) and enolase 1 (ENO1) transcription factor genes can regulate the risk of basal breast cancer^22^. Wu et al. used Fluidigm’s Polaris platform to analyze the heterogeneity of the TNBC cell line SUM149 at the single-cell level for the first time^23^. Breast cancer cells were divided into 5 subpopulations by CSCs and epithelial-mesenchymal transition (EMT)-related genes: EMT CSCs, mesenchymal-epithelial transition (MET) CSCs, dual-EMT-MET CSCs, EMT non-CSCs and non-CSCs. Interestingly, SUM149 is usually classified as triple-negative breast cancer, but its stem cells show heterogeneous expression of marker receptors (ER, PR, and HER2). In addition, these cells exhibit a high degree of heterogeneity in alternative splicing patterns. For example, CSCs show different expression patterns of CD44v6 exons and differential expression of epidermal growth factor receptor (EGFR) transcripts. CD44v6 can promote tumor cell proliferation, while CSCs with high expression of EGFR-211 and EGFR-201 show low invasion and high proliferation features. These results indicate that the heterogeneity of CSCs determines their different proliferation and invasion potential. Emma et al. identified the tumor stem cell subpopulations in the breast cancer cell line MDA-MB-231 and conducted in-depth studies on their biological characteristics^24^. Three breast cancer cell subpopulations were determined via scRNA-seq gene expression profiling and cluster analysis, one of which was a CSC-like subgroup. In addition, pseudotime analysis revealed that these cancer subpopulations have a dynamic process of differentiation from the CSC-like subgroup to the other two groups, confirming that the CSC-like subgroup is the CSC subpopulation. The investigation demonstrated 14 significantly upregulated genes in the CSC-like group, some of which are related to stem cell characteristics and clinical survival data and may be used as potential breast cancer biomarkers to predict and confirm their function, while their role in tumor cells remains to be elucidated^24^. In addition, breast cancer patients with a high risk of recurrence showed higher expression levels of breast CSC markers than breast cancer patients with a low risk of recurrence, illustrating the significance of using breast CSC markers for the prognosis of breast cancer^25^.

In general, potential breast cancer biomarkers related to the characteristics of CSCs have been identified, and other model systems will be used for further experimental verification to confirm their functions and potential clinical applications.

### Heterogeneity of fibroblasts identified by scRNA-seq

In addition, scRNA-seq can be used to analyze the heterogeneity of the components of the breast cancer microenvironment, such as tumor-associated fibroblasts (CAFs) and immune cells^26,27^. CAFs are the main component of the tumor microenvironment. CAFs can promote the proliferation, invasion, metastasis, and immune escape of tumor cells by secreting various cytokines, chemokines, extracellular matrix regulatory molecules, extracellular matrix components, and inflammatory mediators^28^. However, their origin and role in the initiation, development, and treatment response of the disease remain ambiguous.

To improve the classification accuracy of CAFs in breast cancer at the cellular and functional levels, Michael et al. used scRNA-seq to detect mesenchymal cells isolated from tumors in a breast cancer model of MMTV-PyMT mice^26^. Because CAFs do not have a common cell surface marker, a negative screening strategy (EpCAM^−^/CD4^−^/CD31^−^/NG2^−^) was adopted to remove the corresponding epithelial cells, immune cells, endothelial cells, and pericytes in the advanced cancer tissue through fluorescence-activated cell sorting (FACS). Finally, they identified four different cell subpopulations: vascular CAFs (vCAFs), matrix CAFs (mCAFs), cycling CAFs (cCAFs), and developmental CAFs (dCAFs) by functional analysis of gene ontology (GO). The temporal and spatial distribution of cell subsets in the tumor is based on the marker genes of the histological localization. vCAFs originate from vascular pericytes and then invade the tumor matrix area; mCAFs are derived from tissue-resident fibroblasts, cCAFs are the proliferation state of vCAFs, and dCAFs are derived from tumor cells and undergo EMT. In addition, the genetic profile of each CAF subtype is associated with a specific function, and the signature of vCAFs or mCAFs has prognostic power because of its association with metastatic dissemination^26^. Therefore, if the current generalized CAF population resolution is improved, it will enable the development of precisely targeted drugs for CAFs.

### Heterogeneity of immune cells in the tumor microenvironment

Another important component of the tumor microenvironment is immune cells. Their phenotype and characteristics are also vital for understanding tumor progression and immunotherapy. Chung et al. analyzed 175 immune cells from 11 patients (including luminal A, luminal B, HER2, and TNBC patients) and divided the cells into three groups—T lymphocytes, B lymphocytes, and macrophages—according to gene expression profiles at the single-cell level^27^. Both T lymphocytes and macrophages exhibit immunosuppression characteristics. Specifically, T cells have a regulatory or exhausted phenotype, and macrophages have an M2 phenotype. This study used scRNA-seq technology to describe the heterogeneity of immune cells in the tumor microenvironment, but no additional in-depth research has been conducted. The impact of immune cell heterogeneity on the tumor microenvironment warrants more attention.

Although immune cells have remarkable similarities between the normal environment and tumor tissues, Elham et al. observed that immune cells in the tumor microenvironment have a significant expansion of their specific phenotypes^29^. The increased heterogeneity of the intracellular state and apparent phenotypic expansion within the tumor may be due to the diversity of the local microenvironment within the tumor, which differs in its inflammatory levels, hypoxia, activation, and inhibition of receptor-ligand expression, and nutrient supply. By analyzing paired scRNA-seq data and T-cell receptor (TCR) sequencing data, TCR utilization in T-cell phenotypic diversity was discovered^29^. The differences in the TCR clonotype composition and key gene expression of individual T-cell clusters indicate that the phenotypic state may be formed by antigenic TCR stimulation and environmental stimulation^29^. These observations will contribute to a better understanding of the underlying mechanisms by which immune cells promote and resist tumor progression. In addition, Hamad et al. analyzed the gene expression profiles of bone marrow-derived suppressor cells (MDSCs) in the breast cancer model of MMTV-PyMT mice and revealed two different clusters of neutrophils and mononuclear cell lineages in MDSCs (G- and M-MDSCs)^30^. G-MDSCs are produced through the abnormal neutrophil maturation trajectory in the spleen, making them immunosuppressive cells. This study helps to understand the characteristics of MDSCs in breast cancer and their contribution to breast cancer. Therefore, an in-depth study of tumor-infiltrated immune cells provides a way to overcome immune suppression and monitor immune escape in a more illustrative manner.

**Fig. 1:**
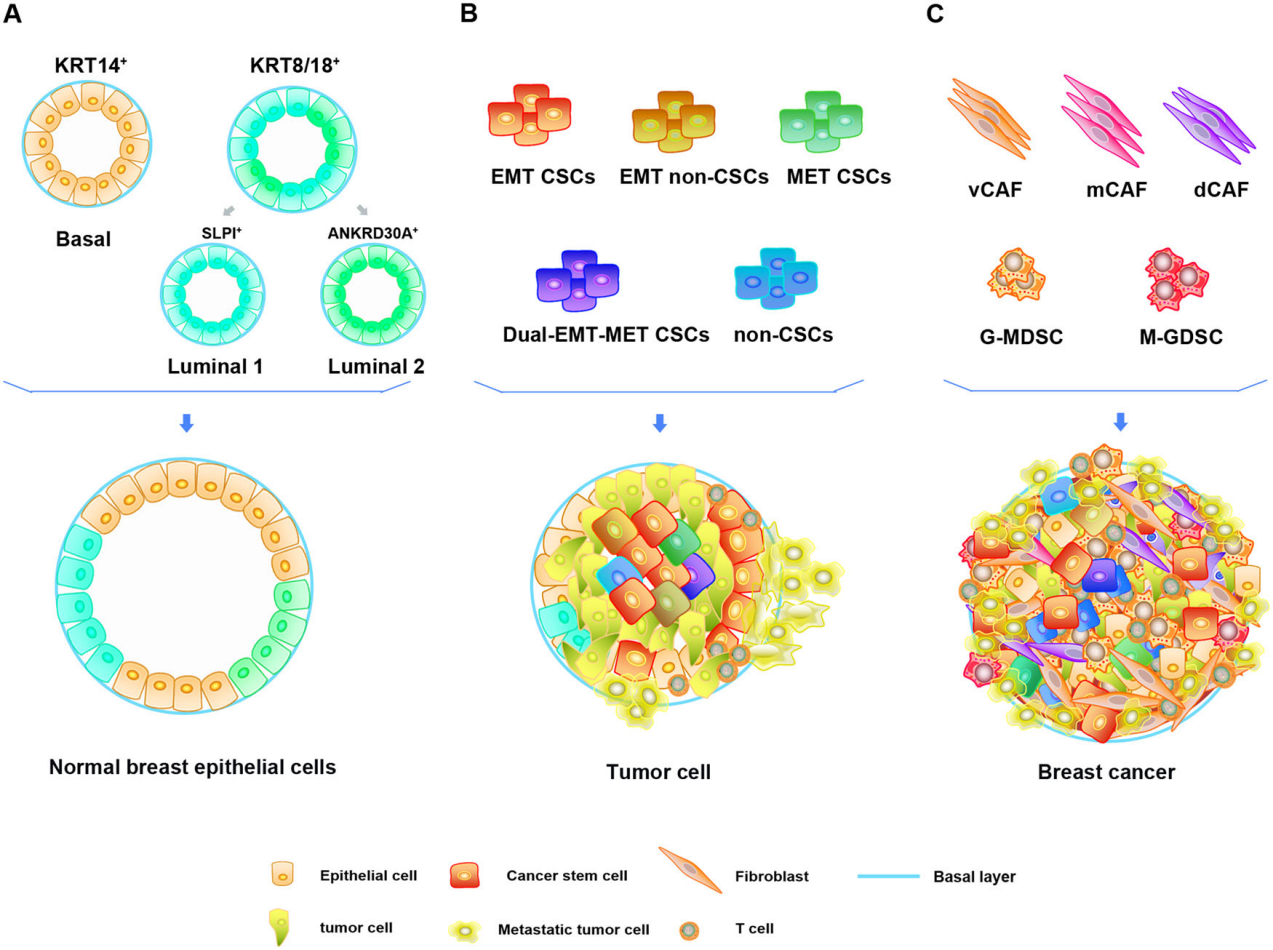
Schematic illustration of the heterogeneity of normal breast epithelium, cancer stem cells, and stromal cells in the tumor microenvironment. **A** The heterogeneity of normal breast epithelial cells. KRT14 and KRT8/18 are genetic markers of basal and luminal breast epithelium, respectively. The luminal epithelial cells are composed of luminal 1 (SLPI^+^) and luminal 2 (ANKRD30A^+^). **B** According to EMT-related genes, cancer stem cells are divided into EMT CSCs, MET CSCs, dual-EMT-MET CSCs, EMT non-CSCs, and non-CSCs. **C** The heterogeneity of tumor-associated fibroblasts and MDSCs in the tumor microenvironment. The tumor-associated fibroblasts at least include the subpopulations of vascular CAFs (vCAFs), matrix CAFs (mCAFs), and developmental CAFs (dCAFs), whereas the MDSCs are identified to be derived from neutrophils (G-MDSCs) and mononuclear cells (M-MDSCs).

In summary, the study of subpopulation identification and features of the heterogeneity of tumor cells and tumor microenvironment cells in breast cancer by scRNA-seq is critical for our understanding of the role of these cells and provides potential new targets for clinical treatment (Fig. 1).

## Identifying the characteristics contributing to breast cancer metastasis by scRNA-seq

### Re-evaluating the EMT using scRNA-seq

The role of EMT in tumor migration and invasion has been widely recognized. Tumor epithelial cells lose polarity and disconnect connections with neighboring cells through EMT while gaining migration and invasion capabilities and showing resistance to apoptosis, in addition to restoring the characteristics of tumor stem cells^31^. To assess the EMT status of single cells, an EMT lineage tracing model (Tri-PyMT) was subjected to scRNA-seq^32^. By comparing pre-EMT and post-EMT cells at the single-cell level, a specific EMT characteristic was determined. This signature contains many EMT markers, such as Vimentin, Fibronectin 1 (Fn1), S100 calcium-binding protein A4 (S100a4), paired related homoeobox 1 (Prrx1), and zinc finger E-box-binding protein 1/2 (ZEb1/2). However, its overlap with the published EMT gene set is limited, suggesting the diversity of EMT programs in different tumors. The study also showed that pre-EMT cells play a leading role in tumor metastasis, while post-EMT cells are not metastasis-initiating cells and secrete more pro-angiogenic factors to support tumor angiogenesis^32^. Importantly, post-EMT cells did not permanently exhibit mesenchymal phenotypes in the Tri-PyMT model and restored the epithelial phenotypes and caused secondary tumors, indicating sustained EMT plasticity. Because single- or small cell subpopulations may cause metastasis, Chen et al.^33^ used a microfluidic device to enrich breast cancer cells with migration ability and scRNA-seq to study the heterogeneity between individual migrating breast cancer cell lines and patient-derived cells. The observed gene expression characteristics clearly distinguished between migratory and wild-type cancer cells for all cell populations. Although all migrating breast cancer cells consistently showed elevated EMT and CSCs, the expression of specific markers of these states were quite heterogeneous. In addition, migrating breast cancer cells showed different gene expression profiles related to oxidative stress, mitochondrial morphology, and proteasome^33^. These cells with EMT characteristics showed unique gene expression distributions, and the new specific genes may be related to the migration and prognosis of breast tumor cells. Yuan et al.^34^ revealed that metastatic tumors contain a higher proportion of EMT markers and cells expressing S100A4 through scRNA-seq. The S100A4 gene related to metastasis is used as a potential diagnostic and therapeutic target. These studies illustrate the significant effect of EMT on breast cancer migration and invasion and can become a new therapeutic target.

### A highly heterogeneous tumor subpopulation possesses enhanced metastasis potential

Another interesting finding elucidated that a subgroup of highly heterogeneous tumors may show enhanced metastasis ability compared with less heterogeneous tumors^35^. This heterogeneity may play a role when the tumor faces strong selective pressures, such as chemotherapy and metastasis barriers. For example, the variable expression of small nuclear ribonucleoprotein 40 (SNRNP40) in spliceosomes promotes the metastasis of tumors. Clinically, subsets with low expression of SNRNP40 are associated with metastasis and recurrence^35^. Michalina et al. also proposed highly heterogeneous tumors with higher metastasis capability. They found that small subclones of breast cancer cells expressing IL11 and vascular endothelial growth factor D (VEGFD) synergistically promoted metastasis, among which scRNA-seq of CD45+ cell populations from primary tumors, blood, and lungs showed that IL11 acts on bone marrow-derived mesenchymal stromal cells and induces pretumorigenic and premetastatic neutrophils to promote the progression of tumor metastasis^36^. In addition, Cai et al.^37^ proposed the chemical probe arginine methyltransferase CARM1 to combat the invasion of breast cancer cells by changing epigenetic plasticity using scRNA-seq. The study revealed that high heterogeneity caused by genetic and epigenetic characteristics of cancer subsets serves as a mechanism to promote tumor metastasis. Overall, scRNA-seq provides a potential reference for studying the development and metastasis mechanism of breast cancer and selects a suitable regimen in real-world clinical practice.

## Application of scRNA-seq in the drug resistance of breast cancer treatment

For nonmetastatic breast cancer, the purpose of treatment is to remove tumors from the breast and regional lymph nodes and avoid metastatic recurrence, including surgery, radiotherapy, chemotherapy (neoadjuvant/adjuvant), ET, targeted therapy, and immunotherapy^7^. For metastatic breast cancer, the therapeutic goals are prolonging life and relieving symptoms. Although clinical treatment regimens are effective for many breast cancer patients, some patients show poor treatment effects due to drug resistance, which reduces overall survival. Presently, the development and molecular mechanism of drug resistance remain unclear.

It remains debatable among scientists whether the emergence of drug resistance results from the selection of pre-existing rare clones or acquisition of new genome mutations. Previously, conducting in-depth research and discussion on this issue was challenging because of the lack of more accurate methods for detecting the genomic information of rare subclones^38^. However, the emergence of scRNA-seq technology provides more possibilities for in-depth exploration of drug resistance mechanisms and further accurate analysis of transcriptome information.

### Pre-existing drug-resistant cells are revealed by scRNA-seq

Through long-term follow-up studies, Samuel et al. conducted an in-depth analysis of the genetic and phenotypic subclonal evolution of four metastatic ER+ breast cancer patients to better understand how breast cancer cells acquire drug resistance at the single-cell level^39^. The results revealed that the tumor cells had a resistant phenotype, which pre-existed in subclones before chemotherapy and showed characteristics related to drug resistance after chemotherapy. The existence of these drug-resistant phenotypes has also been found by Kim’s teams by dissecting 20 TNBC samples during neoadjuvant chemotherapy^8^. These resistant phenotypes were adaptively selected by chemotherapy, accompanied by the reprogramming of the transcriptome, and eventually evolved into a completely resistant phenotype. Moreover, these phenotypes exhibit characteristics associated with drug resistance, such as mesenchymal signal transduction and the enhancement of growth factors, thereby promoting the drug resistance of tumor cells^40^. The downregulation of antigen presentation and TNF-α signaling also makes it easier for tumor cells to escape attack by the immune system^41^. These findings indicate the selection of drug-resistant phenotypes and highlight the ability of cancer phenotypic evolution. These results suggest a phenotype-targeted treatment strategy, which offers new ideas for improving chemotherapy resistance in breast cancer.

### Pre-adapted (PA) cells found by scRNA-seq are essential for the resistant phenotypes of breast cancer

Interestingly, Sung et al. analyzed the drug resistance of ET in luminal breast cancer at single-cell resolution and obtained completely different conclusions^42^. They used scRNA-seq and imaging techniques to analyze the transcriptional variability of plastic cells in tumor cells during ET, and defined a rare PA cell subset in plastic cells. These PA cells are highly enriched in circulating tumor cell clusters and have unique transcriptional signatures, such as dormant characteristics and mixed epithelial and mesenchymal traits. However, it is different from the resistant phenotypes mentioned above^8^. PA cells do not express chemoresistance-related genes—i.e., PA subgroup cells do not show a drug-resistant phenotype. The survival rate of PA cells under acute ET was increased twice compared with that of other plastic cells, while nonplastic cells disappeared completely under selective pressure. After long-term treatment, further transcriptional reprogramming and copy number changes are required to obtain complete drug resistance and further metastasis of breast cancer. Therefore, PA cells are an essential step in achieving drug resistance, but considerable reprogramming is still required to reproduce the characteristics of fully resistant cells^42^. Interestingly, early metastatic cells have been reported to have partially overlapping PA cell characteristics of survival, dormancy, and EMT^43^. PA cells may not only show a survival advantage in the early stages of treatment but also may be a precursor to micrometastases. In addition, the ET multistep resistance model established by Sung et al. may explain the delayed recurrence of patients after ET—i.e., after ET, PA-like cells are adaptively selected and retained for more than 10 years^42^.

**Fig. 2:**
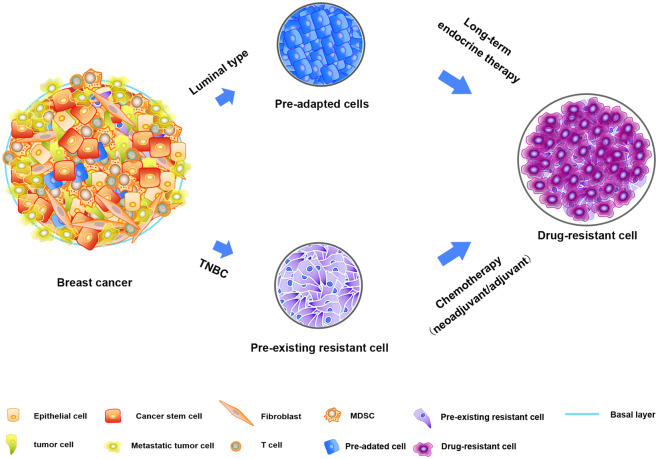
The potential mechanisms of breast cancer cells acquiring drug resistance revealed by the scRNA-seq. The pre-adapted and pre-existing resistant cells exist in the luminal breast cancer and TNBC respectively, and they are expanded contribute to drug resistance after endocrine therapy or chemotherapy.

Overall, the emergence of drug resistance in breast cancer may be caused by pre-existing rare clonal subpopulations. Some of these subpopulations have drug-resistant phenotypes and do not exhibit drug resistance but have survival advantages or other features, such as PA cells (Fig. 2). However, simply summarizing the drug resistance factors as the adaptive selection of clonal subgroups is not sufficient to describe the late recurrence of breast cancer caused by ET^42^. These seemingly contradictory results indicate the complex interaction between genetic and nongenetic factors. Therefore, research on the drug resistance mechanism of breast cancer requires more diversified, in-depth, and comprehensive mining and analysis.

### Characteristics of the immunotherapy response in breast cancer identified by scRNA-seq

In recent years, immune checkpoint inhibitors (ICIs) have been reported to improve the condition of patients with advanced malignancies in clinical trials. However, the efficacy of ICIs is limited to 15–30%, except for melanoma^44^. At the single-cell level, Jang et al.^45^ analyzed the transcriptome and mutation profiles focusing on the tumor mutation burden (TMB), immune checkpoint crosstalk, and radiosensitivity from breast cancer cells and immune cells. They found that, compared with radiotherapy-sensitive cells (RS), a basal subtype, high PD-L1 expression, and high TMB with a mutational signature of microsatellite instability (MSI) are shown by radiotherapy-resistant (RR) cells, while the mutation characteristics of RS cells are mainly gene mutations related to mismatch repair. In addition, in patients with the TNBC or HER2 subtype, the number of immune checkpoint ligand-receptor interactions between tumor and immune cells, such as PD-L1 and CTLA-4, is increased^46,47^. The immune checkpoint crosstalk between tumor and immune cells is related to the subtypes in breast cancer patients^48^. Therefore, for patients who are not sensitive to radiotherapy, ICI therapy can be combined to improve the efficacy. These findings may help determine the potential biomarkers and best combination treatment strategy of immune checkpoints in addition to radiotherapy in breast cancer.

Furthermore, the latest research has found that heme oxygenase-1 (HO-1) can be used as a latent immune checkpoint target in the antitumor immune response caused by chemotherapy. HO-1 is expressed in various cancers as an immune suppressive enzyme, whose activity affects the antitumor response of CD8^+^ T cells in the tumor microenvironment^49^. Tin mesoporphyrin (SnMP) targets both HO-1 and HO-2 as a potent HO inhibitor. James et al. found that SnMP inhibits the immunosuppressive response of HO-1 to CD8^+^ T cells induced by chemotherapy in the tumor microenvironment^49^. In addition, scRNA-seq data showed that HO-1 is mainly derived from the myeloid lineage and is coexpressed with the immune checkpoint PD-L1/2 in human breast tumors. The efficacy of immunostimulatory chemotherapy targeting PD-L1 and HO-1 was compared, proving that SnMP is better than PD-L1 ICIs in preclinical models. Therefore, SnMP can be used as a new immune checkpoint treatment method to improve the sensitivity of the immune system to chemotherapy. Overall, ICIs combined with chemotherapy or radiotherapy are an important strategy for clinical breast cancer treatment in the future.

## Application of scRNA-seq in the prognosis of breast cancer

### CD8^+^ T tissue-resident memory T cells predict a better prognosis in TNBC

Peter et al. performed scRNA-seq of 6311 T cells isolated from human breast cancer. They found a population of CD8^+^ tissue-resident memory T (TRM) cells which highly expressed immune checkpoint molecules and effector proteins^50^. The genetic characteristics of these CD8^+^ TRM cells were significantly related to improving the survival rate of early TNBC patients and had a better prognosis than CD8 expression alone. Therefore, CD8^+^ TRM gene signatures can be used as a marker for a good prognosis of patients.

### The marker genes of BCSC identified by scRNA-seq predict the prognosis of breast cancer

Tong et al. analyzed the gene expression profile of breast cancer stem cells (BCSCs) at the single-cell level and found that the transcriptome of tumor cells is significantly different^25^. Notably, 74 BCSC marker genes were enhanced during the transcription process. Breast cancer patients with a high risk of relapse exhibited higher expression levels of these BCSC markers than those with a low risk of relapse, highlighting the clinical significance of BCSC markers in predicting the prognosis of breast cancer. The 74 identified BCSC markers may become new targets and prognostic markers for breast cancer treatment.

### An alternative polyadenylation (APA) signature predicts the prognosis of breast cancer

In addition to the cellular level, scRNA-seq can also analyze the guidance of posttranscriptional modifications on patient prognosis. APA in 3′ untranslated regions (3′UTRs) is vital in the modification of transcript abundance, localization, and interaction with microRNAs^51^. APA is a posttranscriptional modification of the 3′UTR that affects tumor cell proliferation by adjusting the length of the 3′UTR^52^. It was recently reported to be linked to the prognosis of breast cancer patients^53^. Kim et al. studied the changes in the 3′UTR of breast cancer cells at the single-cell level and found that most breast cancer patients have a shorter 3′UTR^54^. In addition, 3′UTR shortening is closely related to cell proliferation and the dedifferentiation state. They analyzed 10 meaningful genes related to APA that were highly expressed in tumor cells and affected the prognosis of patients. In summary, the expression of APA genes in breast cancer was linked to the clinical outcome of earlier stage breast cancer patients—i.e., the APA signal can be used as a prognostic indicator of early breast cancer.

## Conclusion and perspective

**Fig. 3:**
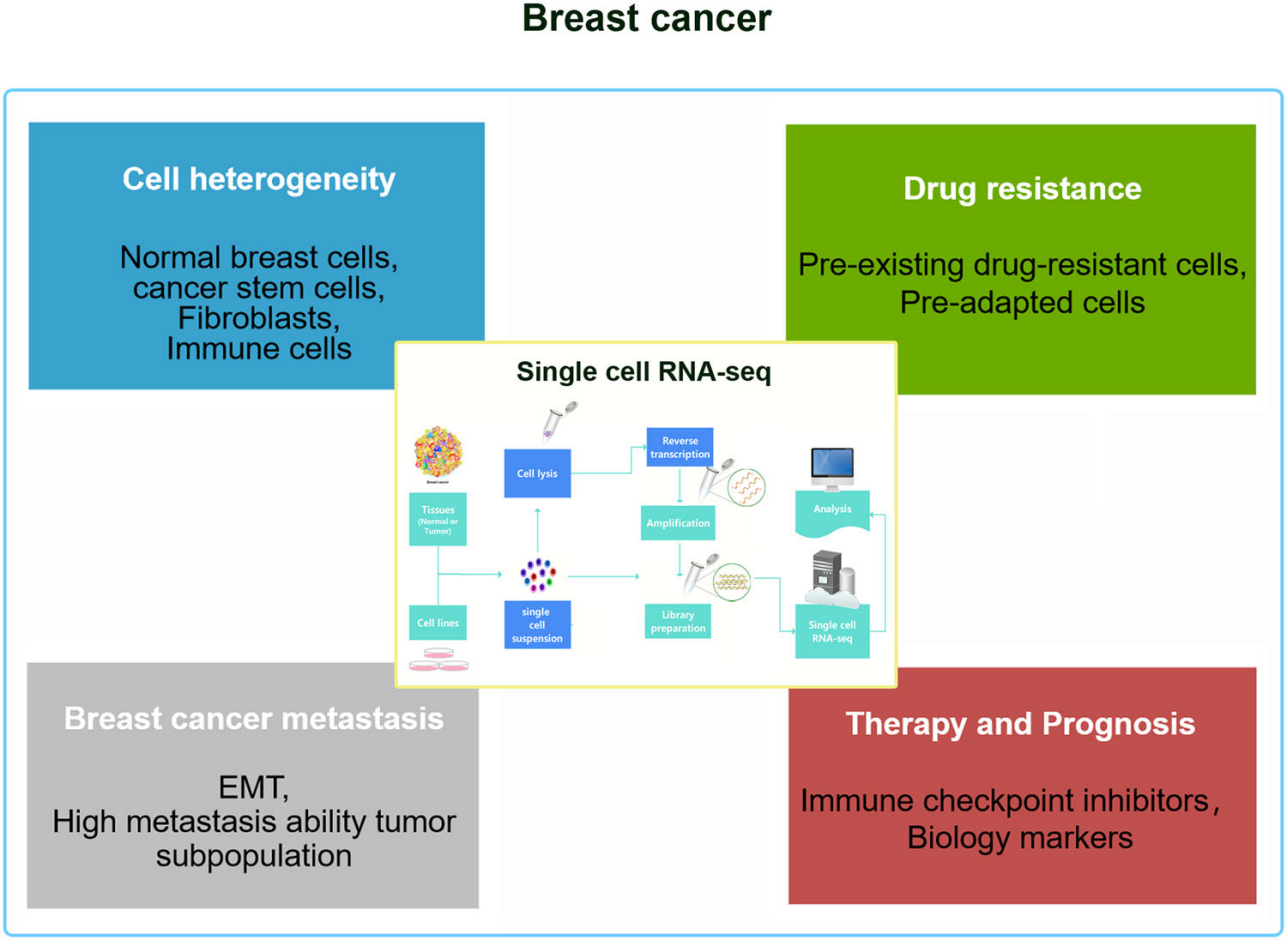
Recapitulation of the contents of the review. Summary of the current application of the scRNA-seq technology in the cell heterogeneity, metastasis, drug resistance, immunotherapy, and prognosis of breast cancer.

As a powerful tool, scRNA-seq technology is convenient to address breast cancer cell heterogeneity, metastasis, drug resistance, breast cancer treatment, and prognosis-related problems (Fig. 3). This opens up a new way to determine new therapeutic targets. However, scRNA-seq technology still faces challenges. The limitations include the high costs and dropout rate, which are often difficult to solve. As the scale and complexity of scRNA-seq data sets increase, faster and more effective computing instruments are needed for processing and analysis. Furthermore, because of incomplete RNA capture and bias/batch effects of PCR amplification on patients or samples, scRNA-seq data sets often contain technical noise sources, which will cause bias in the analysis and interpretation of the data if left unresolved. To meet these challenges, a set of calculation tools has been developed to process, analyze and visualize the scRNA-seq data set to achieve higher resolution clustering and trajectory inference. However, manually annotating cell types with marker genes is very time-consuming. Although new automated and semiautomated methods are being exploited to classify cell types to resolve this problem, new cell types and states must be manually marked. Regarding scRNA-seq, the efficiency of existing technologies, including sensitivity, multiplexing, throughput, and cost-effectiveness, must be improved to develop new technologies.

In conclusion, scRNA-seq technology allows analysis of the single-cell transcriptome with high sensitivity, high precision and high throughput. In addition, the further development of scRNA-seq combined with multiomics will enable obtaining a more comprehensive understanding of cell types and cell states. Combining single-cell transcriptome data and proteome data will help understand how the transcriptome cell state transforms into a functional phenotypic state and the possible heterogeneity at the transcription and translation levels to obtain an in-depth comprehension of tumor evolution. The integration of live cell imaging data and scRNA-seq data could analyze more complex cell phenotypes and spatial positioning and status. In some cases, treatment failure is attributed to the presence of CSCs, which are inherently highly resistant to many treatment methods. Using high-throughput screening technology and systems biology methods can identify new mechanisms of drug resistance and predict molecular markers and genotypes of drug response and involve determining the responsiveness of tumors to specific drug treatments. More importantly, the results of scRNA-seq studies can help design better treatment strategies, such as targeting rare cell populations and highly variable populations, to solve the problem of drug resistance caused by heterogeneity and develop new treatment options. Future research will focus on developing more powerful scRNA-seq technology, which will help unlock the mystery of single cells in various human diseases, provide more cutting-edge data, and show great prospects in biology and clinical treatment for breast cancer and other tumors.
